# Brain Magnetic Resonance Imaging Findings in Patients with Developmental Delay in Addis Ababa, Ethiopia

**DOI:** 10.4314/ejhs.v32i4.14

**Published:** 2022-07

**Authors:** Tewodros Endale Balcha, Abebe Mekonnen Woldeyohannes, Getachew Assefa Neknek

**Affiliations:** 1 Department of Radiology, College of Health Sciences, Addis Ababa University, Addis Ababa, Ethiopia

**Keywords:** Brain, Magnetic resonance imaging, Developmental delay

## Abstract

**Background:**

Developmental delay is a major health problem throughout the world causing significant individual disability. Even though physical examination and patient history are the most important and basic evaluations of patients with developmental delay, additional investigations are usually required in supporting or reaching a diagnosis among which is neuroimaging. This study aims to assess brain Magnetic resonance imaging (MRI) patterns in patients presented with developmental delay.

**Method:**

A retrospective analysis of 164 patients who had undergone brain Magnetic Resonance Imaging (MRI) evaluation for the developmental delay was done. The study was conducted between March to November 2021 G.C at Tikur Anbessa Specialized Hospital (TASH). The patients' clinical history and magnetic resonance imaging reports were reviewed from their medical records. All patients with developmental delay who had brain MRI evaluation at TASH and at one private diagnostic center in Addis Ababa were included in the study.

**Results:**

A total of 164 patients were included in this study of which 95(57.9%) were male and 69(42.1%) female patients were seen. A total of 120 patients (73.2%) showed abnormal brain MRI studies. Previous neurovascular insults were the most common abnormalities seen in 75(45.7%) patients followed by imaging findings of congenital and developmental abnormalities seen in 20(12.2%) patients.

**Conclusion:**

Brain MRI is an important input in the evaluation of patients with developmental delay. It can give evidence for the cause of developmental delay, especially in the diagnosis of perinatal/hypoxic-ischemic insults, and congenital and developmental malformations.

## Introduction

Delay in development occurs when a child fails to attain developmental milestones such as gross and fine motor, speech and language, cognitive and performance, social, psychological, sexual, and activities of daily living as compared to peers from the same population. Global developmental delay (GDD) is defined as a significant delay in two or more of the above developmental domains (1–3).

Although the exact prevalence of developmental delay is unknown, according to WHO 10% of the population in each country population has a disability of one or another kind. The GDD incidence rate is 1% to 3% in schoolage children or younger. Since 95% of the population resides in low and middle-income countries, there is an increased risk for developmental delays and disorders (1,2).

Detailed history and examination are paramount in the assessment of children with global developmental delay (2, 4). But it is frequently not possible to identify the cause of developmental delay through medical and developmental history and physical examination alone (5). Investigations are a useful adjunct in determining etiology. The investigations considered in the literature include genetics, neuroimaging, electrophysiology, and biochemical/metabolic (4). Neuroimaging studies provide important information about central nervous system structure and function, evidence of the previous insult, and characteristic patterns or specific abnormalities that can categorize or identify certain disease processes. Abnormalities detected in neuroimaging studies may direct further investigation even if the diagnosis cannot be reached from imaging alone (5).

Even if there are research works that assessed the causes of developmental delay based on imagings elsewhere, we don't know the imaging patterns in our country. This study assessed the imaging abnormalities seen in patients with developmental delay.

## Methods and Materials

The study was conducted at the Department of Radiology, Tikur Anbessa Specialized Teaching Hospital, Addis Ababa, Ethiopia from March 2021- to November 2021 G.C. The hospital is the largest tertiary level referral and teaching hospital in the country. This teaching hospital is one of the centers of excellence in Ethiopia in undergraduate, postgraduate, and subspecialty programs. The radiology department is one of the many departments in the institution with several experienced radiologists with many years of experience. The department is equipped with high-tech radiologic machines including high-resolution US machines, MDCT machines with 64 and 128 slices, and a high-resolution MR scanner which is Philips Medical Systems Achieva 16 channels 1.5-tesla strength. The one private diagnostic center where brain MRI studies of children were included in the study has a Siemens MRI scanner with 0.35-tesla strength.

A retrospective descriptive study was conducted. A review of clinical data and imaging reports of brain MRI studies of patients who had MRI evaluation for an indication of developmental delay at TASH and at one private diagnostic center was done.

**Source population**: All patients of either gender who were sent to the radiology department of TASH and to one private diagnostic center in Addis Ababa for brain MRI studies

**Study population**: All patients of either gender with clinical data of developmental delay who were sent to the radiology department of TASH and at one diagnostic center in Addis Ababa for brain MRI studies.

A convenient sampling technique was used where all patients of either gender who had brain MRI evaluation for a developmental delay during the period under review at the department of radiology at TASH and at one private diagnostic center in Addis Ababa were included in this study. Patients with brain MRI studies but with other clinical presentations, rather than the developmental delay with/without other additional clinical presentations, were excluded from this study. Brain MR Images were acquired using Philips Medical Systems Achieva 16 channels 1.5 T and Siemens 0.35 T strength MR scanners after making the child sleep or sedated. The brain MR images were interpreted by senior consultant neuroradiologists with experience ranging from 2 to more than 10 years of experience and fellows in neuroradiology. Brian MR imaging findings were recorded on a data collecting format that was designed to include patients' sex, age, clinical information, and imaging findings.

**Data processing and analysis**: The data was cleaned, coded, and entered into SPSS version 26. Frequencies, mean, standard deviation, percentages, and cross-tabulation were determined and summarized. Association studies between variables were done using the chi-square test (using an α value of <0.05 as a statistically significant association).

**Ethical considerations**: Data collection was started after getting permission from the ethical review committee of the department of radiology at Addis Ababa University. Patient confidentiality was maintained by omitting patients' names and hospital identification numbers from the data collecting format and selected representative medical images.

## Results

From a total of 164 patients included in this study, 95(57.9%) were male and 69(42.1%) were female patients with the age range of 5 months to 13 years, with a mean of 3 ± 2.5 years. The majority of patients were seen in the age range of 1–2 years and 2–5 years, accounting for 62(37.8%) and 70(42.7%) patients respectively.

**Figure 1:**
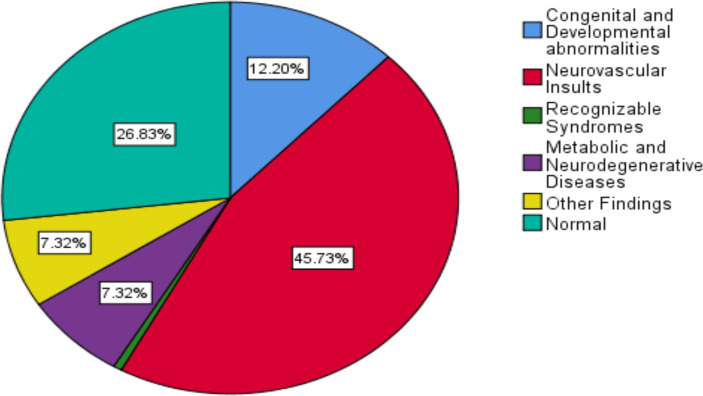
Frequency of final imaging diagnosis in 164 patients with developmental delay who came for brain MRI evaluation at TASH and at one private diagnostic center in Addis Ababa in 2019–21

A total of 120 patients (73.2%) showed abnormal brain MRI studies, with 70(58.3%) male and 50(41.7%) female patients, while the rest 44 (26.8%) patients showed normal studies. Abnormalities of previous neurovascular insults were the most common findings seen in 75(45.7%) patients followed by congenital and developmental abnormalities, seen in 20(12.2%) patients (Figure 1).

Features of metabolic and neurodegenerative abnormalities and the category of other imaging findings, which included non-specific focal or global brain parenchymal volume loss and non-specific white matter abnormalities account for 12(7.3%) cases each. Imaging feature of tuberous sclerosis was seen in one patient under the category of recognizable syndromes.

Among the total 164 patients, 35 patients presented with additional clinical findings such as seizure, microcephaly, neurologic deficits, perinatal history of asphyxia, etc., in addition to developmental delay. A total of 33(94.3%) patients from the 35 showed abnormal brain MRI findings which are a higher percentage when compared to those presented with developmental delay alone, accounting for 87(67.4%) out of 129 cases. This difference showed statistical significance, with a p-value of 0.001.

**Figure 2:**
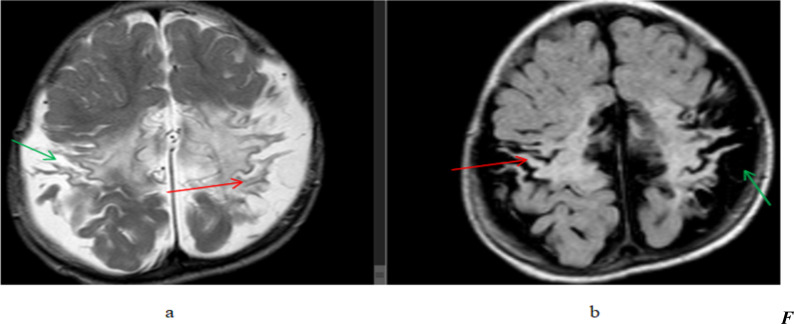
A 1-year-old male patient presented with developmental delay and a history of perinatal asphyxia, (a) T2W axial and (b) FLAIR axial, brain MR images showing bilateral parasagittal frontoparietal(perirolandic region) thinned gyri with hyperintense signal change(gliosis)(red arrows) and widening of sulci and subarachnoid CSF spaces(green arrows). Findings are consistent with the HIE sequela

From 75(45.7%) cases out of the 164 patients who showed abnormalities of previous neurovascular insult, hypoxic-ischemic encephalopathy (HIE) was seen in 69(42.1%) cases (Figure 2). The remaining 6(3.6%) cases were previous neurovascular occlusion (infractions). Imaging diagnosis of previous neurovascular insults was the most common abnormality in both genders and all age groups (Table 1).

**Table 1:** Distribution of imaging diagnosis among age ranges in 164 patients with developmental delay who came for brain MRI evaluation at TASH and at one private diagnostic center in Addis Ababa in 2019–21

		Final Imaging Diagnosis						Total
		Congenital and Developmental abnormalities	Neurovascular Insults	Recognizable Syndromes	Metabolic and Neurodegenerative Diseases	Other Findings	Normal	
Age range of patients in years	<1 yr	1 8.3%	4 33.3%	0 0.0%	2 16.7%	1 8.3%	4 33.3%	12 100.0%
	1–2 yr	6 9.7%	36 58.1%	0 0.0%	4 6.5%	3 4.8%	13 21.0%	62 100.0%
	2–5 yr	12 17.1%	29 41.4%	0 0.0%	4 5.7%	5 7.1%	20 28.6%	70 100.0%
	>5 yr	1 5.0%	6 30.0%	1 5.0%	2 10.0%	3 15.0%	7 35.0%	20 100.0%

**Figure 3:**
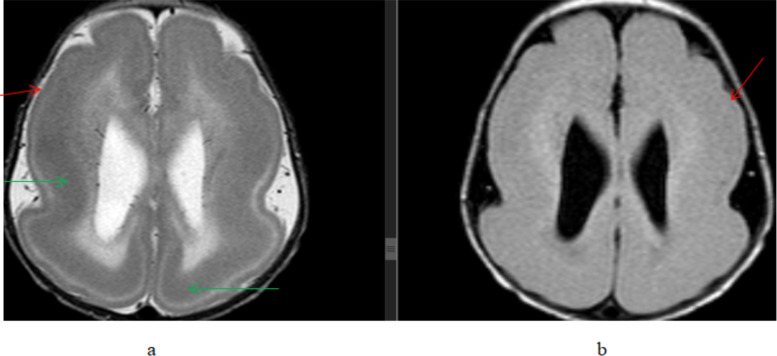
An 8-month-old female patient presented with developmental delay and seizure, (a) T2W axial and (b) FLAIR axial, brain MR images showing bilateral cerebral hemisphere smooth surface outline(red arrows) with a subcortical band of T2 hypointensity(green arrow). Findings are consistent with lissencephaly with band heterotopia

**Figure 4:**
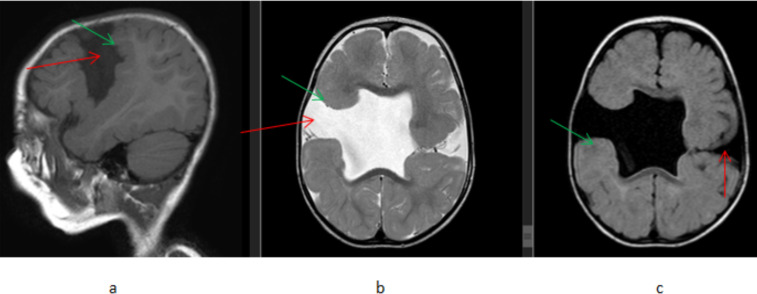
A 1-year-old male patient presented with developmental delay and seizure, (a) T1W sagittal (b) T2W axial and (b) FLAIR axial, brain MR images showing bilateral posterior frontal lobe CSF filled clefts(red arrows) which are grey matter lined(green arrows) that extend from surface to the body of lateral ventricles. There is also an absent septum pellucidum. Findings are consistent with bilateral Schizencephaly

Congenital and developmental abnormalities were seen in 20(12.2%) patients with cortical malformation accounting for the majority of the cases, 15 cases. These cortical malformations include 11 cases of lissencephaly/pachygyria/polymicrogyria, 3 cases of schizencephaly, and 2 cases of heterotopia (Figure 3 and Figure 4). Corpus callosum agenesis was seen in 4 patients. One case of holoprosencephaly was seen. One patient showed more than one anomaly, which is corpus callosum agenesis and heterotopia.

**Figure 5:**
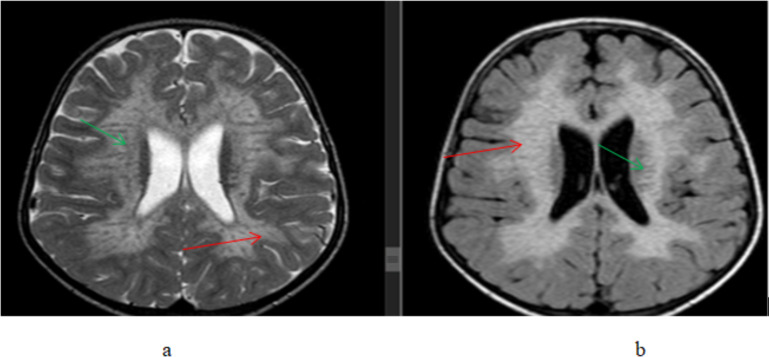
A 3-year-old female patient presented with developmental delay, (a) T2W axial and (b) FLAIR axial, brain MR images showing bilateral periventricular white matter hyperintense signal changes (red arrows) with sparing of subcortical U fibers and internal linear hypointensities(green arrows) giving “tigroid pattern”; The imaging features are those of metachromatic leukodystrophy

Imaging features of metabolic and neurodegenerative diseases were seen in 12(7.2%) patients, with 10 cases of bilateral basal ganglia T2/FLAIR hyperintensities and 2 cases of bilateral cerebral diffuse white matter T2/FLAIR hyperintensities (Figure 5).

Only one case of a possible neurocutaneous syndrome, tuberous sclerosis, was seen in a 9-year-old male child with clinical data of developmental delay with brain MRI findings of multiple T1 hypointense and T2 hyperintense bilateral cerebral subcortical foci of signal changes.

Those abnormalities and imaging findings that are not included in the above categories were summarized under other findings, accounting for 12(7.3%) cases. These include 7 cases of non-specific focal or global brain parenchymal volume loss, 4 cases of non-specific T2/FLAIR white matter hyperintensities, and one case of ventricular dilation.

## Discussion

This study assessed brain MRI of 164 patients who presented with developmental delay and showed abnormal brain MRI findings in the majority of the patients, 120 (72.3%), which is a similar finding to previous studies done in India by T Arul Dasan and Deepashree B, and Mathew A. et al showing 72% and 75% of abnormal brain MRI findings respectively (6, 7). But similar studies done in Iran by Momen et al and in Egypt by Tolba S. Abd Elghaffar et al showed a prevalence of 58.6% and 82% respectively(8,9). These variations could be explained possibly by variations in source populations.

The percentage of patients with abnormal brain MRI findings was higher in those who presented with additional clinical data such as seizure, microcephaly, neurologic deficits, perinatal history of asphyxia, etc. compared to those with developmental delay alone, showing statistical significance. Previous studies and literature stated a similar higher yield of brain MRI in patients with developmental delay and additional clinical findings (3, 7, 10, 11).

The most common abnormal brain MRI finding in this study was an imaging pattern of previous neurovascular insults, accounting for 75(45.7%) patients. Out of which, sequela of hypoxic-ischemic encephalopathy (HIE) was seen in 69(42.1%) patients. This finding, in terms of the most common abnormality, is similar to the previous two studies done in India which showed 60% cases of HIE and 58.8% cases of ischemic sequela (6,7). But a study done in Egypt by Tolba S. Abd Elghaffar et al. showed metabolic and degenerative findings to be the most abnormal findings seen in 41.5% of patients (9). This could be explained by a difference in study populations.

Imaging findings of congenital and developmental abnormalities were the second most common abnormalities in this study seen in 20(12.2%) patients, with 15 patients showing cortical malformations such as lissencephaly/pachygyria, polymicrogyria, schizencephaly, and heterotopia. This finding is similar to previous studies by Althaf Ali S. et al and Kalaiarasan et al showing a frequency of 17% and 12% respectively under the category of congenital and developmental abnormalities (10, 11).

Imaging findings that are suspicious for metabolic/neurodegenerative diseases were seen in 12(7.2%) of patients in this study with a similar percentage to a study in Iran by Momen et al (8). But this diagnosis was the most common abnormality in a study done in Egypt by Tolba S. Abd Elghaffar et al accounting for 41.5% of the cases (9). This difference could be explained by the study population difference as previously mentioned.

Under the category of other findings, accounting for 12(7.3%) patients, imaging findings of 7 cases of non-specific focal or global brain parenchymal volume loss, 4 cases of nonspecific T2/FLAIR white matter hyperintensities, and one case of ventricular dilation were included. The parenchymal volume losses were suggested as a possible sequela of traumatic or infectious insult for which previous clinical history correlation was recommended in these patients. The non-specific white matter hyperintensities were suggested as the possibility of delayed myelination or terminal zone of myelination and follow-up imaging was recommended.

This study has many limitations. This study didn't include additional detailed clinical data of patients such as detailed perinatal history, including gestational age, mode, and circumstances of child delivery, which are very important inputs in guiding the assessment of brain imaging of children with developmental delay. It was better if other tests, such as metabolic, electroencephalographic, and genetic studies, were part of the study in selected patients, to help guide or correlate with the imaging findings. It was also important if follow-up imaging were available in some selected patients to see any temporal change in some of the imaging findings, such as the non-specific white matter hyperintensities and basal ganglia abnormalities. As this is a retrospective study where all images couldn't be retrieved from the archives, rather we used image reports, which is also one of the limitations of the study.

In conclusion brain MRI is an important input in the evaluation of patients with developmental delay. Brain MRI can give evidence for the cause of developmental delay, especially in the diagnosis of perinatal/hypoxic-ischemic insults, and congenital and developmental malformations, which are the most common abnormalities seen in this study. Brain MRI can also reveal features of metabolic and neurodegenerative changes in patients with developmental delay in which further diagnostic studies could be suggested. The yield of brain MRI in patients with developmental delay is increased in those who have additional clinical findings, such as seizure and neurologic deficit.
